# Case report: Reactive nodular fibrous pseudotumor: report of 4 new cases including a rare pulmonary presentation and a literature review covering 40 cases

**DOI:** 10.3389/fmed.2026.1891307

**Published:** 2026-08-03

**Authors:** Xinyao Liu, Chenghui Wang, Weiran Kong, Bangdong Liu, Tianlun Li, Susu Shi, Linlin Zhang, Shuai Chen

**Affiliations:** Department of Pathology, Affiliated Hospital of Jining Medical University (Clinical school of medicine), Jining, China

**Keywords:** benign lesion, clinicopathological features, fibrous pseudotumor, immunohistochemistry, reactive nodular fibrous pseudotumor

## Abstract

Reactive nodular fibrous pseudotumor (RNFP) is a rare benign fibrocollagenous lesion that often poses diagnostic challenges because of its nonspecific clinical and radiological features, particularly at unusual sites such as the lung. This study aimed to characterize the clinicopathological, immunohistochemical, and prognostic features of RNFP and summarize the literature.

We retrospectively analyzed four pathologically confirmed RNFP cases, including a rare pulmonary case, and combined them with 40 reported cases (n = 44). RNFP occurred at multiple anatomical sites, most commonly in the abdomen, followed by the extremities and chest. The pulmonary lesion presented as a solitary nodule mimicking malignancy.

Histologically, all lesions showed low cellularity, abundant hyalinized collagen, and scattered bland spindle cells without cytologic atypia or mitoses. Immunohistochemically, all cases were positive for vimentin; 50% expressed SMA and 25% showed focal CD34 expression, whereas STAT6, β-catenin, S-100, and desmin were consistently negative. All molecular detection results were uniformly negative. During follow-up (6–60 months), no recurrence or progression was observed.

RNFP is a benign reactive lesion with characteristic histological and immunophenotypic features. Diagnosis relies on morphological assessment and immunohistochemical exclusion of mimics. Awareness of its anatomical distribution, including rare pulmonary involvement, is essential to avoid misdiagnosis and overtreatment.

## Introduction

Reactive nodular fibrous pseudotumor (RNFP) is a rare benign fibrocollagenous lesion characterized by localized nodular proliferation of dense collagen accompanied by sparse spindle-shaped fibroblasts or myofibroblasts. Since its first systematic description by Yantiss et al. (1), RNFP has been regarded as a reactive, non-neoplastic lesion that most commonly

arises in the mesentery and subserosal tissues of the gastrointestinal tract. Although benign, RNFP often presents as a localized mass that can clinically and radiologically mimic various neoplastic lesions, leading to potential misdiagnosis as an invasive tumor or metastatic disease.

Previous studies have shown that RNFP predominantly occurs in the gastrointestinal serosa, mesentery, and related intra-abdominal structures, whereas involvement of other anatomical sites is rare. In particular, RNFP arising in the lung has rarely been reported in the literature. Therefore, reporting and summarizing cases in such unusual locations can help expand the known anatomical distribution of RNFP and enhance awareness among clinicians and pathologists.

In this study, we report four cases of RNFP diagnosed at our institution, including an extremely rare pulmonary case. Through a detailed clinicopathological analysis of these cases combined with data from previously published literature, we aim to further delineate the anatomical spectrum of RNFP, summarize its key clinicopathological features, and highlight important considerations for differential diagnosis, thereby facilitating accurate recognition and appropriate clinical management.

## Materials and methods

### Patients and samples

Following approval by the Institutional Review Board, the pathology archives of our institution were retrospectively searched for cases of reactive nodular fibrous pseudotumor (RNFP) diagnosed between January 2015 and January 2026. A total of four cases met the inclusion criteria and were included in the study. Clinical data were retrieved from the medical records, including patient demographics, presenting symptoms, imaging findings, lesion location, surgical management, and gross pathological characteristics.

All available hematoxylin and eosin (H&E) - stained slides were independently reviewed by two experienced pathologists to confirm the diagnosis according to established histopathological criteria. Cases with equivocal histological features or insufficient tissue for evaluation were excluded.

### Immunohistochemistry

Immunohistochemical staining was performed on formalin-fixed, paraffin-embedded (FFPE) tissue sections using standard laboratory protocols. The antibody panel included vimentin (V9), smooth muscle actin (SMA, E8V5B), desmin (MX046), CD34 (MX123), β-catenin (MXR022), S-100 protein (MXR034), and Ki-67 (MX006) (all from Fuzhou Maixin Biotech Co., Ltd., China).

Appropriate positive and negative controls were included in each staining run. Cytoplasmic and/or membranous brown staining was considered indicative of positive expression for vimentin, SMA, desmin, and CD34. S-100 expression was assessed based on cytoplasmic and/or nuclear staining, whereas β-catenin and Ki-67 expression were evaluated on the basis of nuclear staining.

All immunohistochemical results were interpreted in conjunction with the histomorphological findings to support the diagnosis and facilitate the exclusion of potential histologic mimics.

### Molecular genetic analysis

Formalin-fixed paraffin-embedded (FFPE) tumor tissue samples were collected from the four patients diagnosed with RNFP. Genomic DNA was extracted from FFPE sections using a commercial tissue DNA extraction kit according to the manufacturer's instructions.

Targeted mutation analysis was performed using a fluorescence-based quantitative real-time PCR assay with a commercially available five-gene mutation detection kit (Amoy Diagnostics Co., Ltd., Xiamen, China). The assay was designed to detect hotspot mutations in commonly altered solid tumor–associated driver genes, including *ALK, ROS1, EGFR, KRAS, NRAS, BRAF, RET, HER2, MET*, and *PIK3CA*.

Briefly, DNA concentration and purity were assessed by spectrophotometry. Qualified DNA samples were then mixed with PCR master mix, primers, and fluorescent probes provided in the kit. Real-time PCR amplification was performed on a real-time PCR instrument following the standardized thermal cycling protocol recommended by the manufacturer.

Amplification curves and cycle threshold (Ct) values were interpreted according to the manufacturer's instructions. Each run included appropriate positive and negative controls to ensure assay validity.

### Literature review

A comprehensive literature review was conducted to identify all available published reports of reactive nodular fibrous pseudotumor (RNFP). The literature search was conducted using the PubMed, Google Scholar, and China National Knowledge Infrastructure (CNKI) databases and covered publications published between January 2003 and April 2026. The final database search was completed on April 11, 2026.

The search strategy included the terms “reactive nodular fibrous pseudotumor,” “reactive nodular fibrous tumor,” “fibrous pseudotumor,” “fibrous pseudotumour,” and “RNFP,” which were searched individually and in various Boolean combinations across the selected databases. No language restrictions were applied, and publications in both English and Chinese were eligible for inclusion.

All retrieved records were independently screened by two reviewers. The selection process consisted of an initial screening of titles and abstracts, followed by a full-text review of potentially eligible articles.

The inclusion criteria were as follows: (1) histopathologically confirmed RNFP and (2) availability of sufficient clinical, pathological, and follow-up information. The exclusion criteria were as follows: (1) duplicate publications or overlapping case reports; (2) cases with insufficient or missing essential clinical or pathological information; and (3) cases lacking definitive diagnostic confirmation.

For patients reported in multiple publications, only the most comprehensive report was retained for analysis to minimize duplication bias.

Data extracted from each eligible study included patient age, sex, lesion location, clinical presentation, histopathological features, follow-up duration, and recurrence status.

Based on the present search strategy, no previously reported cases of primary pulmonary RNFP were identified. Nevertheless, this finding should be interpreted with caution because of the rarity of the disease and the potential limitations of database indexing and literature retrieval.

## Case

### Case 1 (Lung)

A 55-year-old woman was incidentally found to have bilateral pulmonary nodules during a routine health examination 2 years earlier and subsequently underwent wedge resection of a nodule in the left lung. Follow-up chest computed tomography (CT) demonstrated enlargement of a nodule in the right middle lobe, which was subsequently resected. Intraoperatively, the lesion exhibited ill-defined borders and was not associated with pleural retraction. Gross examination revealed a firm nodule measuring 1.5 × 1.3 × 1.3 cm with a gray-white cut surface located adjacent to the pleura.

Histologically, the lesion was composed predominantly of abundant hyalinized collagen containing scattered spindle-shaped to stellate fibroblasts, sparse lymphocytic infiltrates, and focal calcification, without significant cytologic atypia or identifiable mitotic activity. Immunohistochemically, the spindle cells showed positivity for vimentin and SMA and were negative for S-100, CD117, ALK, desmin, and CD34. The Ki-67 labeling index was approximately 2%.

Taken together with the characteristic histomorphological findings, the immunophenotypic profile supported a diagnosis of pulmonary reactive nodular fibrous pseudotumor (RNFP).

### Case 2 (Appendix)

A 55-year-old man presented with persistent dull abdominal pain, and imaging findings suggested a possible appendiceal mucocele. His medical history was notable for left lower limb fracture repair 5 years earlier. Intraoperatively, a firm, well-circumscribed mass measuring 5 × 5 cm was identified in the mid-appendix, and an appendectomy with partial cecal resection was performed.

Gross examination revealed a firm lesion measuring 7 × 6 × 5 cm with a gray-white to gray-yellow cut surface, attached to an 8 cm segment of the appendix. Microscopically, the subserosal lesion showed a low-cellularity spindle cell proliferation within abundant dense collagen, accompanied by features of acute suppurative appendicitis.

Immunohistochemically, the spindle cells were positive for vimentin and negative for CD34, S-100, CD117, DOG1, SMA, desmin, ALK, and β-catenin. The Ki-67 labeling index was approximately 1%.

A diagnosis of appendiceal reactive nodular fibrous pseudotumor (RNFP) with concomitant acute suppurative appendicitis was established.

### Case 3 (Stomach)

A 72-year-old man with a history of sigmoid colon carcinoma presented with a 6-month history of a protruding lesion in the gastric antrum. Gastroscopy revealed a firm, poorly mobile submucosal lesion measuring 0.6 cm on the anterior wall of the gastric antrum, and endoscopic submucosal dissection was subsequently performed.

Gross examination revealed a specimen measuring 1.0 × 0.9 × 0.2 cm and containing a firm gray-white nodule measuring 0.5 × 0.5 cm. Histologically, the lesion was composed of a low-cellularity spindle cell proliferation embedded within abundant densely hyalinized collagen.

Immunohistochemically, the spindle cells showed focal positivity for CD34 and were negative for SMA, desmin, CD117, DOG1, and S-100. The Ki-67 labeling index was approximately 1%−2%.

Taken together with the characteristic histomorphological findings, the immunophenotypic profile supported a diagnosis of gastric reactive nodular fibrous pseudotumor (RNFP).

### Case 4 (Greater omentum)

A 46-year-old woman with a history of right oophorectomy and myomectomy presented with dysmenorrhea and menorrhagia of 6 months' duration. Pelvic ultrasonography revealed multiple hypoechoic uterine nodules and a mass within the lower uterine cavity, consistent with uterine leiomyomas. A total hysterectomy was performed, and multiple nodules were incidentally identified in the greater omentum during surgery. Consequently, an omentectomy was also performed.

Gross examination revealed an omental specimen measuring 9 × 4 × 1.8 cm and containing multiple gray-white nodules, the largest measuring 0.5 × 0.4 × 0.2 cm. Histologically, the nodules were composed of hypocellular fibrous proliferations with abundant collagen deposition and scattered bland spindle cells, without significant cytologic atypia or identifiable mitotic activity.

Immunohistochemically, the lesional cells showed diffuse vimentin immunoreactivity and partial SMA staining, whereas they were entirely negative for β-catenin, CD34, S-100 and desmin. Rare scattered Ki-67-labeled nuclei were observed, consistent with a markedly low proliferative index.

Based on the characteristic histomorphological and immunophenotypic findings, a diagnosis of omental reactive nodular fibrous pseudotumor (RNFP) was rendered (Table 1).

**Table 1:** Immunohistochemical results of 4 cases.

Immunohistochemical marker	1 (Lung)	2 (Appendix)	3 (Stomach)	4 (Greater omentum)
vimentin	+	+	+	+
CD34	–	–	Partially positive +	–
S−100	–	–	–	–
CD117	–	–	–	–
Dog−1	–	–	–	–
SMA	+	–	–	Partially positive +
desmin	–	–	–	–
ALK	–	–	–	–
β-Catenin	–	–	–	–
Ki−67	1% (+)	1% (+)	1–2% (+)	Scattered (+, < 1%)

## Results

A total of 44 cases of reactive nodular fibrous pseudotumor (RNFP) were included in this study, including 4 cases from our institution and 40 cases identified from the published literature. The clinical characteristics, lesion distribution, tumor size, histomorphological features, and immunohistochemical profiles of all cases were summarized and descriptively analyzed (Table 2).

**Table 2:** Clinical features of Reactive nodular fibrous pseudotumor (*n* = 44).

Reference	Age	Sex	Location	Maximum diameter (cm)	Clinical features	Past medical history	Follow–up
Case 1	55	F	Lung	1.5	Hard gray–white mass	Left segmentectomy performed 2 years ago	NED−21mo
Case 2	55	M	Appendiceal serosa	7	Firm gray–white mass	Left lower leg fracture 5 years ago	NED−60mo
Case 3	73	M	The anterior wall of the gastric antrum	0.5	Gray–white mucosal elevation	Sigmoid colon cancer resection 4 years ago	NED−29mo
Case 4	46	F	Greater omentum	0.5	Gray–white nodule	Right oophorectomy and myomectomy performed 10 years ago	NED−20mo
Ismini et al. (1)	80	M	Sigmoid colon		Gross–solid mass	Left inguinal hernia for 20 years	NED−36mo
Qiusheng Xiong et al. (2)	42	F	Lesser curvature of the stomach	2.5	Gray–white central elevation	Long–term habit of dieting and high–intensity abdominal workouts	NED−12mo
Ondrej et al. (3)	59	M	Small bowel	3	–	Gastric resection	Four patients under follow–up showed NED within 7 years
Ondrej et al. (3)	46	M	Sigmoid colon		White–fibrous mass	An iron pin found within the tumorous tissue		
Ondrej et al. (3)	1	M	Appendiceal	3	Fibrous lesion	–		
Ondrej et al. (3)	68	M	Cecum	10	Ill–defined mass	–		
Ondrej et al. (3)	30	F	Small bowel	8	–	Chronic bowel obstruction		
Ondrej et al. (3)	65	F	Large bowel	8	Yellow–white, elastic mass	–		
Ondrej et al. (3)	22	M	Ileum	7	Hard–fibrous mass	–		
Ondrej et al. (3)	41	M	Sigmoid colon	6	–	–		
Junfa Chen et al. (4)	54	M	Splenogastric space		Moderately firm, pale–red mass	Laparoscopic radical distal gastrectomy	NED−28mo
Dongxue Wang et al. (5)	62	F	Ileum	5	–	Right oophorectomy and appendectomy performed 2 years ago	–
Liming Wang et al. (6)	54	M	Rectum	4.9	Firm, gray–yellow mass	Pelvic mass present for 2 years	–
Jinesa et al. (7)	65	F	Serosa of the stomach	1.8	Pale, hard, brownish perigastric nodule	Resection of gastric gastrointestinal stromal tumor	–
Jun Liu et al. (8)	53	M	Ileocecal region	6	Moderately firm, gray–white mass	–	–
Jiangyi Xiao et al. (9)	16	F	Stomach	8	Rough–surfaced mass	–	NED−24mo
Xiaoning Gao et al. (10)	49	F	Uterus	13	Gray–white, encapsulated mass	G5P1	–
Birgul et al. (11)	71	M	Between diaphragm, transverse colon, and stomach	19.5	Brownish–gray encapsulated mass	Colon cancer resection	–
Liping Fu et al. (12)	64	M	Stomach	6	Solid–soft tissue mass	Gastrectomy	NED−5mo
Rawand et al. (13)	45	F	Greater omentum, sigmoid colon, and right ovary	7	Hard–white mass	Refractory abnormal uterine bleeding	No evidence of recurrence
Susan et al. (14)	65	M	Mesentery	3	Brown–white mass	–	–
G. Gauchotte et al. (15)	60	M	Gastric antrum	2.2	Hard, gray–white mass	Gastritis of the gastric antrum and duodenal ulcer	NED−4mo
Richa Todi et al. (16)	20	F	Stomach, distal pancreas, and spleen	20	Well–defined soft tissue mass	–	–
Fei Yan et al. (17)	60	F	Mesentery, greater omentum, and colonic serosa	10	Rubbery, hard, white–brown nodule	Myomectomy	NED−12mo
E. Arzu Saglam et al. (18)	28	F	Ovary, appendix, etc.	6	Hard, gray–white nodule	Endometriotic cyst	–
Edoardo Virgilio et al. (19)	71	M	Mesentery	6	Hard–white mass	Left hemicolectomy	NED−48mo
Yantiss et al. (20)	48	M	Mesentery	6.5	Well–defined, brownish–yellow or gray fibrous mass	Recurrent pancreatitis	NED−10~26mo
Yantiss et al. (20)	50	F	Pancreas	4.3		Multiple endocrine neoplasia type 1		
Yantiss et al. (20)	53	M	Mesentery etc.	5.5		–		
Yantiss et al. (20)	57	M	Ileal serosa and mesentery	6.5		–		
Yantiss et al. (20)	71	M	Transverse colon	2.8		Laparoscopic cholecystectomy and gastrinoma resection		
Saulino et al. (21)	20	M	Stomach	5.6	Lobulated gray–white mass	Suspected abdominal wall hernia or rectus diastasis several years earlier.	NED−12mo
Qi Zhe et al. (22)	40	M	Small intestine and mesentery	2.0	Nodular, firm mass	–	NED−14mo
Tingting Huang et al. (23)	38	F	Mesentery	10.6	Firm, well–circumscribed nodule	History of total hysterectomy with bilateral adnexectomy 3 years ago	–
Ho–Man Tam et al. (24)	54	F	Hepatic hilum	2.0	An infiltrative mass	History of laparoscopic appendectomy and laparoscopic cholecystectomy more than 10 years ago	–
Jarod et al. (25)	13	F	Anti–mesenteric jejunal mass	8.8	Firm, gray mass	–	–
Linhon Song et al. (26)	38	M	Mesentery	2	Gray–white, firm mass	History of subtotal gastrectomy 1 year ago	–
Linhon Song et al. (26)	41	F	Intraperitoneal	7	Gray–white, firm mass	Myomectomy for uterine leiomyoma 3 years ago	NED−11mo
Linhon Song et al. (26)	59	M	Posterior to the rectum	4	Gray–white, firm mass	History of prostatectomy	NED−29mo
Linhon Song et al. (26)	54	M	Stomach	5	Gray–white, firm mass	Abdominal discomfort for more than 10 years	–

### Demographic characteristics

RNFP predominantly affects middle-aged and older adults. Patients aged 41–60 years and 61–80 years accounted for 51.1% and 25.7% of all cases, respectively, together representing 76.8% of the cohort. This age distribution may be related to aging-associated biological processes, including impaired tissue repair capacity and increased susceptibility to chronic inflammatory stimuli, both of which have been implicated in fibrosis-related pathogenesis across various tissues (27). Regarding sex distribution, males were affected slightly more frequently than females (59.1% vs. 40.9%), yielding a male-to-female ratio of approximately 1.44:1. This difference may reflect the influence of sex-related biological factors and systemic host–environment interactions, including differences in inflammatory regulation; however, the underlying mechanisms remain unclear (28). Nevertheless, these hypotheses remain speculative given the limited number of reported cases.

### Anatomical distribution

**Figure 1:**
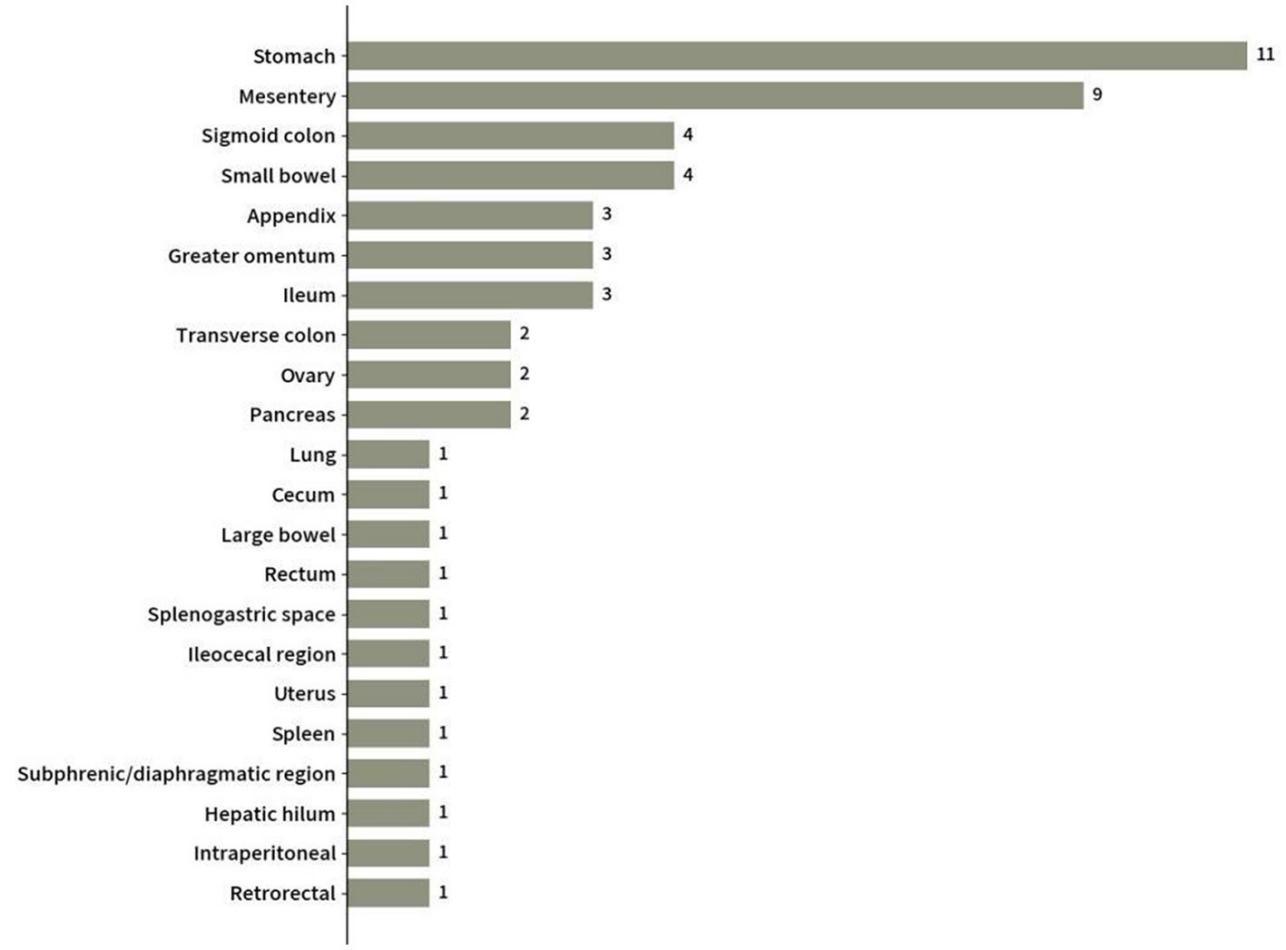
Anatomical distribution of RNFP in 44 cases.

With respect to anatomical distribution, RNFP most commonly involves the digestive system, accounting for 65.7% of all reported cases. The stomach and mesentery were the most commonly involved sites, followed by the sigmoid colon, appendix, greater omentum, and ileum (Figure 1). This distribution pattern may be related to the gastrointestinal tract's long-term exposure to mechanical irritation, luminal contents, and microbial metabolites, which may contribute to repeated tissue injury and persistent reparative responses.

Notably, one pulmonary case of RNFP was identified in the present study, suggesting that this lesion is not confined to the digestive system. The patient had a history of pulmonary segmentectomy, raising the possibility that local chronic inflammation and postoperative tissue repair contributed to the development of the lesion. This observation further supports the hypothesis that RNFP is a reactive proliferative lesion associated with tissue injury and repair and may occur in multiple organ systems.

### Lesion characteristics and clinical implications

RNFP lesions exhibited a wide size range (0.5–20.0 cm), with 69.6% measuring 2–10 cm. The mean and median sizes were 6.08 cm and 6.0 cm, respectively. These findings highlight the importance of individualized clinical management. Small, asymptomatic lesions with benign imaging features may be managed with close observation, whereas larger or symptomatic masses often require surgical resection because malignancy cannot be reliably excluded based on clinical and radiological findings. Grossly, most lesions appear as gray-white, firm masses with abundant collagen deposition, which differs from the gray-red, soft appearance typical of many malignant tumors.

In the present cohort, more than half of the patients had a history of prior surgery at the primary or adjacent site, suggesting that local tissue injury and postoperative reparative processes may contribute to the development of RNFP. Chronic inflammatory stimulation may also be involved in its pathogenesis, further supporting the concept that RNFP represents a reactive, rather than true neoplastic, lesion. However, because clinical and imaging findings are largely nonspecific, a definitive diagnosis still relies on histopathological evaluation.

### Histopathologic features

**Figure 2:**
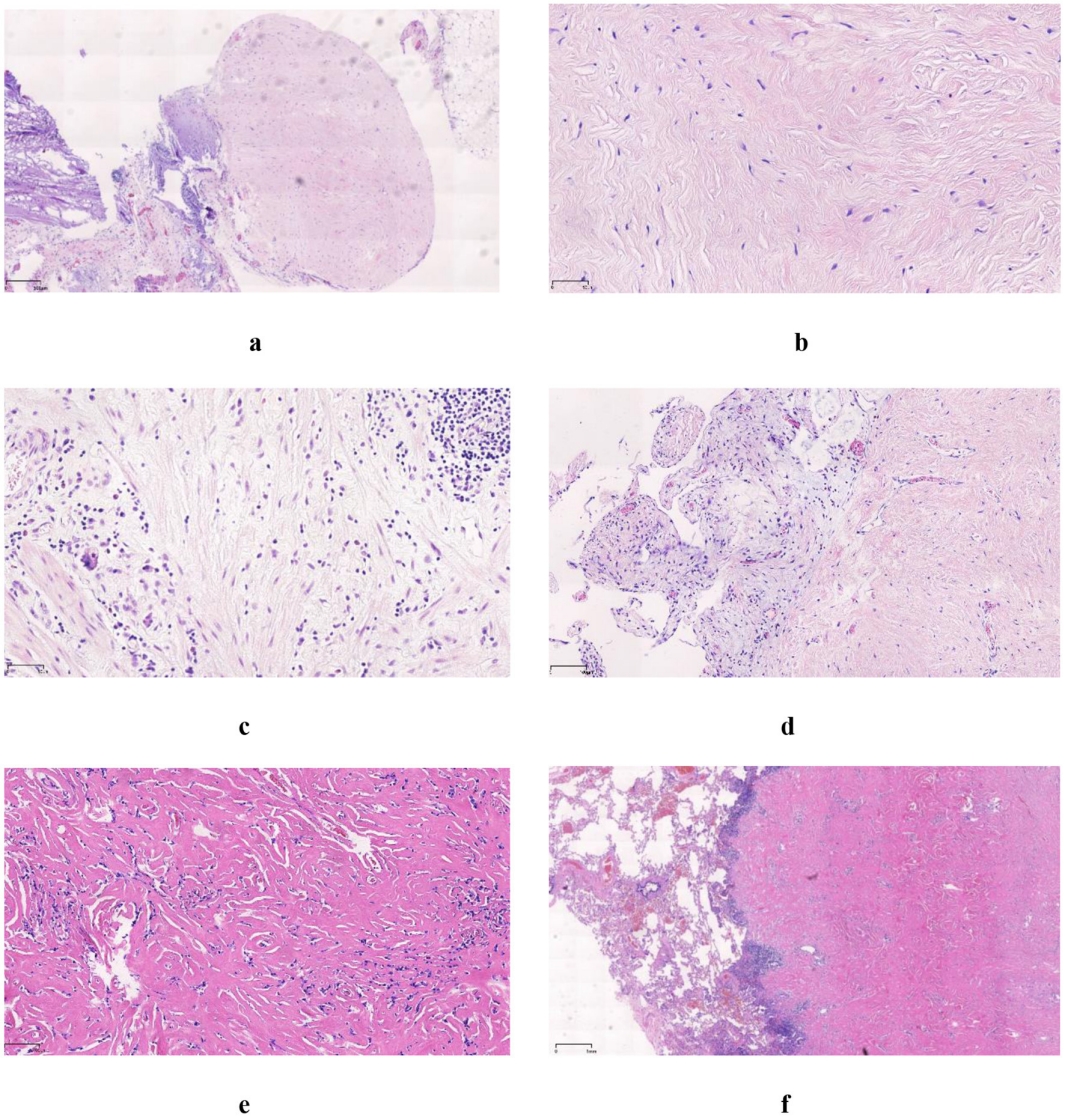
Histopathological features of reactive nodular fibrous pseudotumor (RNFP). **(a)** At low magnification, a nodular lesion composed of dense collagenous stroma is observed (H&E, × 2); **(b, c)** At higher magnification, sparsely distributed spindle-shaped fibroblasts and myofibroblasts are embedded within abundant hyalinized collagen, without evident nuclear atypia (H&E, × 10 and × 20); **(d)** Involvement of the serosa is observed (H&E, × 10); **(e, f)** Histological morphology of pulmonary RNFP (H&E, × 10 and × 2).

Histologically, RNFP is characterized by a well-circumscribed nodular or tumor-like fibrocollagenous proliferation (Figure 2a). The lesion is predominantly composed of abundant dense collagenous stroma, frequently exhibiting hyalinization or keloid-like collagen deposition. Within this collagen-rich stroma, scattered spindle-shaped or stellate fibroblasts and myofibroblasts are present (Figure 2b). These cells are arranged in short fascicles, intersecting bundles, or loose aggregates, resulting in the characteristic hypocellular appearance of the lesion.

The lesional cells exhibit bland cytomorphologic features, including elongated to oval nuclei, inconspicuous nucleoli, and pale eosinophilic cytoplasm. Cytologic atypia is absent, and mitotic activity is either exceedingly rare or entirely absent. Tumor necrosis is not observed (Figure 2c).

The stroma frequently contains variable inflammatory infiltrates composed predominantly of lymphocytes and plasma cells. These inflammatory cells may be diffusely scattered or form focal aggregates, and occasional lymphoid follicle formation may also be observed. Additional histological findings may include focal myxoid change, coarse scar-like collagen deposition, dystrophic calcification, mast cell infiltration, and, occasionally, foreign body–type multinucleated giant cells.

RNFP may involve the serosa, mesentery, omentum, or muscularis propria (Figure 2d) and may exhibit transmural extension, whereas the mucosa is typically spared. Collectively, these histomorphological features support the reactive and non-neoplastic nature of RNFP and provide important diagnostic clues for distinguishing it from other fibrous and myofibroblastic lesions of the gastrointestinal tract and soft tissues.

Cases derived from our pulmonary tumor cohort also presented as relatively well-demarcated nodules microscopically (Figure 2e). The lesions were composed of abundant dense collagenous stroma with hyalinization, where bland spindle and stellate fibroblasts and myofibroblasts were scattered throughout the collagen-rich stroma (Figure 2f).

### Immunohistochemical features

Immunohistochemical studies have demonstrated a relatively consistent immunophenotypic profile across reported RNFP cases (Table 3). The spindle and stellate cells typically show diffuse positivity for vimentin, supporting their mesenchymal origin. Smooth muscle actin (SMA) is expressed in most cases, either diffusely or focally, indicating variable degrees of myofibroblastic differentiation. In contrast, desmin is usually negative and only rarely shows focal, weak positivity.

**Table 3:** Immunohistochemical marker expression in patients.

Immunohistochemistry	No. patients (+)/total no. of patients assessed with marker
vimentin	30/30
CD34	4/36
SMA	29/37
desmin	8/32
CD117	10/35
CK	1/18
AE1/AE3	9/12
MSA	12/18
CaM	1/8
NF	1/8
ER/PR	1/1
CD3	1/1
L26	1/1
S-100	0/34
Dog-1	0/14
ALK	0/27
β-Catenin	0/7
P63	0/3
Calretinin	0/2
EMA	0/9
Syn	0/2
Keratin	0/5
CD31	0/1
D2-40	0/1
PDGFR	0/1
Ki-67	1-2%

In addition to these markers, RNFP generally lacks expression of lineage-specific markers. Gastrointestinal stromal tumor–associated markers, including DOG1, are consistently negative, whereas CD117 (KIT) is negative in most cases but has been reported to be positive in a small number of cases. Neural markers, such as S-100 protein, synaptophysin, calretinin, and neurofilament (NF), are also typically absent. Similarly, epithelial markers, including pan-cytokeratin (AE1/AE3), epithelial membrane antigen (EMA), and p63, are negative in most cases.

CD34 expression shows some variability across reported cases but is most often negative or only focally and weakly positive. In addition, STAT6 and nuclear β-catenin expression are generally absent. The proliferative activity, as assessed by Ki-67, is typically low, with labeling indices in most cases ranging from approximately 1%−2%.

**Figure 3:**
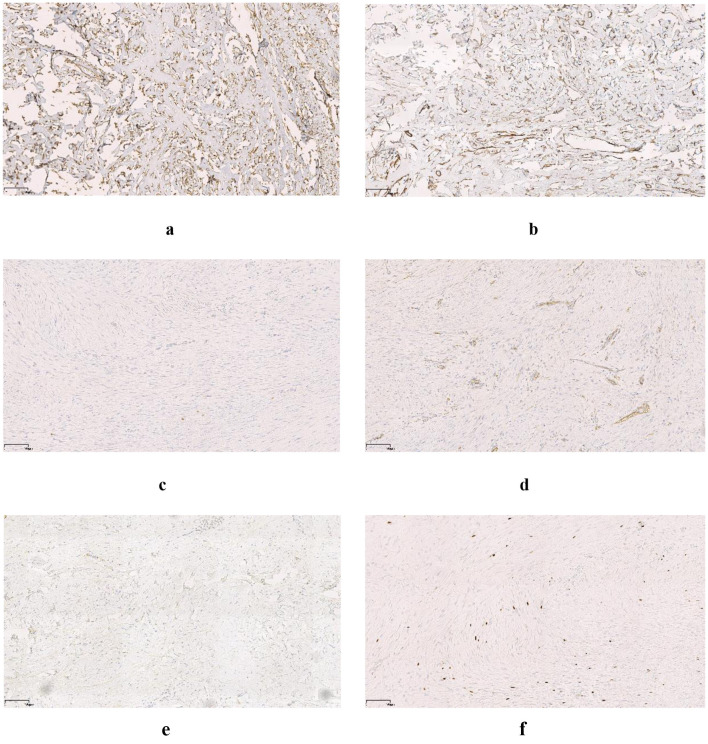
Immunohistochemical findings of the RNFP lesion in our hospital( × 10). **(a)** Positive expression of vimentin; **(b)** Positive expression of SMA; **(c)** Negative expression of CD117; **(d)** Negative expression of β-catenin; **(e)** Negative expression of desmin; **(f)** Low Ki-67 proliferation index.

Overall, RNFP displays a characteristic immunophenotype, showing diffuse vimentin positivity (Figure 3a), frequent SMA immunoreactivity (Figure 3b), and loss of expression of lineage-specific markers, including CD117 (Figure 3c), β-catenin (Figure 3d), and desmin (Figure 3e), together with a low proliferative index (Figure 3f). These findings were largely in keeping with those observed in our cohort (Figure 3). When interpreted in conjunction with its characteristic histological features, this immunoprofile provides important support for the diagnosis of RNFP.

### Molecular genetic results

**Figure 4:**
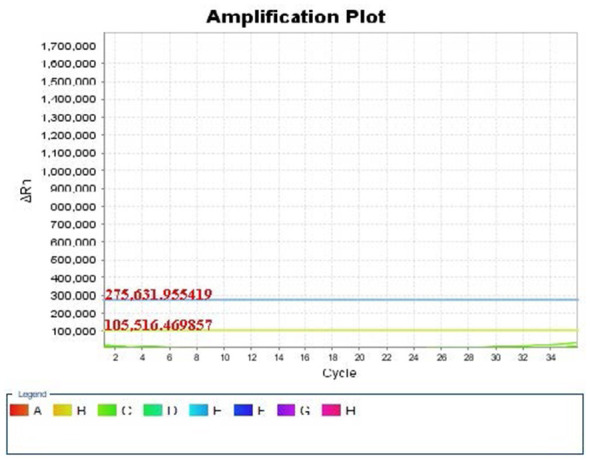
Quantitative real-time PCR amplification plots of gene mutation analysis in RNFP cases. Amplification plots of real-time PCR analysis showing no detectable amplification signals in RNFP cases for all tested genes.

Molecular analysis revealed no detectable mutations in all four cases. No amplification curves were detected for any of the tested genes (*ALK, ROS1, EGFR, KRAS, NRAS, BRAF, RET, HER2, MET, and PIK3CA*) in all four RNFP cases (Figure 4). All Ct values exceeded the predefined positive cut-off thresholds, and no pathogenic hotspot mutations were identified.

These uniformly negative findings indicate the absence of common solid tumor–associated driver mutations in RNFP and further support its benign, non-neoplastic nature. They also aid in distinguishing RNFP from morphologically similar spindle cell or epithelial neoplasms.

### Prognosis and follow-up

Follow-up data further support the benign clinical behavior of RNFP. Among cases with available follow-up data, all patients remained free of disease during the follow-up period. The mean follow-up duration was 21.0 months, and the longest documented follow-up period was 60 months, during which no recurrence was observed. These findings indicate that RNFP is generally associated with a favorable prognosis. Nevertheless, continued clinical follow-up may be considered, particularly during the early postoperative period, given the limited number of reported cases and the relatively short follow-up duration in some patients. RNFP generally exhibits an indolent clinical course and favorable prognosis. Complete surgical excision appears curative in most reported cases. No metastatic behavior or disease-related mortality has been documented to date. Recurrence is exceedingly rare, and patients with available follow-up data have remained disease-free after treatment. No metastatic behavior or disease-related mortality has been reported in the literature to date. Complete surgical excision appears to be curative in most patients.

## Differential diagnosis

The diagnosis of reactive nodular fibrous pseudotumor (RNFP) relies on the recognition of its characteristic histomorphological features, including a hypocellular fibrocollagenous proliferation composed of bland spindle and stellate cells embedded within densely hyalinized collagen, together with a characteristic immunohistochemical profile. Because its clinical and radiological manifestations are often nonspecific, distinction from a wide variety of benign and malignant spindle cell lesions is essential.

Particular attention should be paid to pulmonary lesions, as RNFP occurring in the lung is exceptionally rare and may be easily mistaken for neoplastic processes. In this setting, the principal differential diagnoses include solitary fibrous tumor (SFT), inflammatory myofibroblastic tumor (IMT), calcifying fibrous tumor (CFT), scar-like or inflammatory nodules, and primary or metastatic malignancies. SFT typically exhibits a patternless architecture with branching staghorn-like vessels and shows diffuse CD34 and nuclear STAT6 positivity resulting from the NAB2–STAT6 fusion (29). In contrast, RNFP lacks characteristic staghorn vasculature and is negative for nuclear STAT6, with CD34 expression being absent or only focally positive. IMT is characterized by a cellular myofibroblastic proliferation accompanied by prominent inflammatory infiltrates and frequently demonstrates ALK expression (30), whereas RNFP lacks neoplastic myofibroblastic proliferation and is negative for ALK. CFT shares the presence of dense hyalinized collagen but is typically associated with prominent dystrophic or psammomatous calcifications and a more conspicuous lymphoplasmacytic infiltrate (31), features not typically observed in RNFP. Scar-like or inflammatory nodules typically lack the well-circumscribed nodular architecture and organized collagen bundles characteristic of RNFP and are often associated with a history of infection, trauma, or prior surgical intervention (32). Primary or metastatic malignancies typically exhibit cytologic atypia, increased mitotic activity, necrosis, and infiltrative or destructive growth patterns, features that are absent in RNFP (33).

In the gastrointestinal tract and soft tissues, additional consideration should be given to gastrointestinal stromal tumor (GIST) and desmoid-type fibromatosis (DTF). GIST is composed of spindle and epithelioid cells arranged in fascicular or nested patterns and characteristically expresses CD117 (KIT) and DOG1 (34). Although CD117 positivity has been reported in a small number of RNFP cases, previous studies have shown that this expression is not associated with *c-kit* gene mutations (3). DTF demonstrates infiltrative growth of uniform spindle cells arranged in long fascicles and shows nuclear β-catenin expression (35). These morphological and immunohistochemical features are not observed in RNFP. SFT, IMT, and CFT should also be considered in these anatomical sites and can be excluded using the same diagnostic criteria described above.

Overall, although RNFP often exhibits characteristic histological features, accurate diagnosis requires integration of morphology with immunohistochemical findings and careful exclusion of relevant mimics. Recognition of this entity is important to prevent misdiagnosis as a spindle cell neoplasm or malignancy and to avoid unnecessary overtreatment.

## Discussion

This study reports four cases of pathologically confirmed reactive nodular fibrous pseudotumor, including one case arising in the lung, which is extremely rare and, to the best of our knowledge, has not been previously documented at this anatomical site. This observation further broadens the reported anatomical spectrum of this lesion (17).

Although reactive nodular fibrous pseudotumor is widely regarded as a benign reactive process, it may still represent a diagnostic pitfall in clinical practice, particularly at uncommon anatomical sites such as the lung, where it can mimic neoplastic lesions on imaging and gross examination, potentially leading to preoperative misinterpretation (4). In this context, the present study combines institutional cases with a literature review to emphasize the diagnostic challenges at rare anatomical locations and to discuss their implications for clinical management.

When occurring in the lung, RNFP may show clinical and radiological features overlapping with those of various pulmonary spindle cell lesions, which may complicate preoperative assessment. In the present case, imaging revealed a well circumscribed solitary pulmonary nodule, a feature that can also be observed in solitary fibrous tumor (SFT) (36). In addition, a history of prior pulmonary fibrous and inflammatory changes may have contributed to subtle alterations in local tissue architecture and may have interfered with radiological interpretation, resulting in nonspecific imaging findings that mimic neoplastic lesions. Therefore, differential diagnosis from primary lung cancer and inflammatory mass forming lesions is essential.

A review of the literature further indicates that most cases are incidentally detected and lack specific clinical manifestations, which may increase the risk of clinical misinterpretation (15). Accordingly, a definitive diagnosis cannot be established based on clinical and radiological findings alone and requires histopathological evaluation. These observations suggest that reactive nodular fibrous pseudotumor may be considered in the differential diagnosis of pulmonary nodules with atypical but seemingly benign radiologic features, and tissue sampling may be considered when appropriate.

Based on our limited case series and the available literature, the diagnosis of RNFP relies on the integration of characteristic histopathological features with supportive immunohistochemical and clinical information. Key histopathological findings include a well-circumscribed, paucicellular nodular lesion composed of densely collagenized or hyalinized stroma with scattered bland spindle cells. The consistent absence of cytologic atypia, mitotic activity, and infiltrative growth represents a crucial diagnostic feature.

Supportive immunohistochemical findings are nonspecific and are primarily useful for excluding other entities. These include variable expression of vimentin and smooth muscle actin, along with negative staining for STAT6, ALK, S-100, DOG-1 and β-catenin. These results aid in distinguishing RNFP from the major spindle cell neoplasms in the differential diagnosis.

Additional supportive features may include a history of prior surgery, trauma, or inflammatory conditions in a subset of cases. Available follow up data suggest an indolent clinical course after complete excision, although the limited number of cases precludes definitive conclusions. Negative molecular testing for common oncogenic drivers such as *ALK, ROS1, EGFR, KRAS, NRAS, BRAF*, and *PIK3CA* may provide additional supportive evidence in selected cases.

Recognition of the reactive nature of this lesion is important for clinical management. Both the present series and previously reported cases suggest that complete local excision appears sufficient for disease control, and neither extended resection nor adjuvant therapy is required. This indolent behavior supports the concept that reactive nodular fibrous pseudotumor may represent an exaggerated reparative response to local injury, inflammation, or ischemia rather than a true neoplastic process with autonomous proliferative capacity (13). Accordingly, additional aggressive treatment is unnecessary after diagnosis, which may help avoid overtreatment due to diagnostic uncertainty (9).

Although this study provides a systematic summary of RNFP based on a case series and literature review, several limitations should be acknowledged. First, the retrospective design and limited case number preclude meaningful statistical analysis. Second, the heterogeneity of previously published reports introduces potential bias in the literature-derived data. With the accumulation of additional cases, particularly from rare anatomical sites, a more refined understanding of the pathogenesis of RNFP may be achieved, including whether it represents a distinct entity or a shared end-stage reparative process following diverse forms of tissue injury.

## Conclusion

RNFP is a rare benign fibrocollagenous lesion with characteristic histopathological features and an overall favorable clinical behavior. This study expands its recognized anatomical spectrum by documenting a rare case of pulmonary involvement. RNFP should be considered in the differential diagnosis of mass-forming lesions across multiple anatomical sites. Diagnosis requires correlation of morphological features with immunohistochemical findings to avoid misinterpretation and overtreatment.

## Data Availability

The original contributions presented in the study are included in the article/supplementary material, further inquiries can be directed to the corresponding author.
